# Deep Neural Network for Visual Stimulus-Based Reaction Time Estimation Using the Periodogram of Single-Trial EEG

**DOI:** 10.3390/s20216090

**Published:** 2020-10-27

**Authors:** Mohammad Samin Nur Chowdhury, Arindam Dutta, Matthew Kyle Robison, Chris Blais, Gene Arnold Brewer, Daniel Wesley Bliss

**Affiliations:** 1 School of Electrical, Computer & Energy Engineering, Arizona State University, Tempe, AZ 85281, USA; adutta7@asu.edu (A.D.); d.w.bliss@asu.edu (D.W.B.); 2 Department of Psychology, The University of Texas at Arlington, Arlington, TX 76019, USA; matthew.robison@uta.edu; 3 Department of Psychology, Arizona State University, Tempe, AZ 85281, USA; crblais@asu.edu (C.B.); gene.brewer@asu.edu (G.A.B.)

**Keywords:** EEG signal processing, deep learning, Convolutional Neural Network (CNN), Fully Connected Neural Network (FCNN), periodogram, brain–computer interface, reaction time

## Abstract

Multiplexed deep neural networks (DNN) have engendered high-performance predictive models gaining popularity for decoding brain waves, extensively collected in the form of electroencephalogram (EEG) signals. In this paper, to the best of our knowledge, we introduce a first-ever DNN-based generalized approach to estimate reaction time (RT) using the periodogram representation of single-trial EEG in a visual stimulus-response experiment with 48 participants. We have designed a Fully Connected Neural Network (FCNN) and a Convolutional Neural Network (CNN) to predict and classify RTs for each trial. Though deep neural networks are widely known for classification applications, cascading FCNN/CNN with the Random Forest model, we designed a robust regression-based estimator to predict RT. With the FCNN model, the accuracies obtained for binary and 3-class classification were 93% and 76%, respectively, which further improved with the use of CNN (94% and 78%, respectively). The regression-based approach predicted RTs with correlation coefficients (CC) of 0.78 and 0.80 for FCNN and CNN, respectively. Investigating further, we found that the left central as well as parietal and occipital lobes were crucial for predicting RT, with significant activities in the *theta* and *alpha* frequency bands.

## 1. Introduction

Thanks to advancements in sensor and hardware capabilities, and signal processing techniques, we now have better tools to understand the brain. Cognitive and affective state monitoring has become a topic of interest in understanding various sensory-motor functions [1]. Accurate prediction of reaction time (RT) using neurophysiological biomarkers can help detect various mental states and develop better human–computer interfaces for patients and healthy subjects. This paper focuses on analyzing human response towards visual stimulus and provides methods of estimating RT from information embedded in electroencephalogram (EEG) signals [2]. Our study not only is useful to understand psychological phenomenology but also helps in the translation of brain signals into machine-comprehensible commands that can facilitate augmentative and alternative communication (AAC) using the brain–computer interface (BCI) [3]. Most BCI applications favor EEG as the tool to infer the mental state and to understand communicative intent [4]. One of the pervasive challenges in BCI research is the reduced BCI accuracy over long sessions due to fluctuations in mental state [5,6,7]. Thus, by accurately predicting RT, the mental state can be more accurately assessed, yielding a better BCI accuracy. Accurate prediction of RT can help determine lapses in attention or vigilance, the onset of drowsiness or fatigue, decline of motivation, etc., which can help assess human performance, especially in critical tasks such as the air-traffic control or long-haul driving [3,8]. People with a wide range of language and speech impairments, including congenital impairments such as autism and cerebral palsy [9] as well as acquired conditions such as spinal muscular atrophy (SMA) [10] and amyotrophic lateral sclerosis (ALS) [11] can be largely benefited by this. In addition to this, it can also help monitor people with neuromuscular disorders and in stroke rehabilitation [12,13,14,15].

As reactions stimulated during perceptual decision-making vary considerably from trial-to-trial, it is a significant challenge to predict RT based on subtle and intricate information extracted from EEG signals. In this study, we used the periodogram of single-trial EEG data as the feature space, which is one of the most well-defined forms of spectral information representation. Moreover, we designed and implemented state-of-the-art models based on Fully Connected Neural Network (FCNN) and Convolutional Neural Network (CNN) to learn from the periodogram features and to predict RT. We performed binary and 3-class classification as well as regression using the models. These models are becoming more popular as they can be implemented on field-programmable gate arrays (FPGA) accelerators with high performance. This study shows promise from a computational perspective as well as demonstrates improvement of the prediction performance by a fair margin compared to relevant earlier works. We further tried to interpret the phenomenology by localizing relevant EEG channels and frequency bands that play the most significant role in predicting RT.

## 2. Background

A number of studies have been conducted to analyze the relationship between visual events and corresponding brain signals. EEG frequency and reaction time-based sequential analysis was performed in one of the prior studies to establish a relationship between speed of response and background frequency [16]. Another study demonstrated an experiment to monitor EEG signal and reaction time during a normoxic saturation dive. Three of the Navy divers were exposed to a normoxic breathing mixture at a certain pressure for seven consecutive days. Visual evoked responses (VERs), electroencephalograms (EEGs), and simple reaction time (RT) were measured for all. The outcomes of the experiment indicate changes in the amplitude of both alpha and theta, with a decrease in alpha frequency [17]. One of the studies tried to apply a method for temporally extracting stimulus and response-locked components of brain activity from human scalp electroencephalography (EEG) during an auditory simple reaction time task [18]. A power spectrum-based analysis was performed using Independent Component Analysis (ICA) and Principal Component Analysis (PCA) to estimate visual attention from the EEG signal [19]. Twenty-six pre-elite table tennis players in Taiwan were recruited to perform a cued reaction time task to observe the relation between reaction time and EEG signal [20]. Another comprehensive study showed efforts to evaluate human performance based on features extracted from EEG signals [21].

However, the number of investigations is significantly lower when it comes to directly estimate RTs based on EEG signals. One of the contemporary studies revealed a method to extract Riemannian tangent space features from EEG data and to use these features to estimate RT across 16 participants who completed the task [22]. The study evaluated root mean square error (RMSE) and correlation coefficient (CC) for RT estimation of individual subjects. The resulting average RMSE was 132.5 ms, and the average CC was 0.61. Another study [23] focused on determining the timing of target onset during rapid serial visual presentation based on single-trial EEG data. This investigation utilized data from only six subjects and achieved an average RMSE of 119.5 ms for individual RT estimations. These studies are representatives of attempts to predict RT from EEG data using subject-specific, regression-only estimation models. The subject-specific predictive models have a good impact on study of the human brain, but they lack significance when it comes to practical implementation as they are trained and tested for individual subjects. Also, regression-only models limit the scopes, leaving cases where low-computation and high-precision categorical information regarding human response is needed. Our previous work [24] tried to overcome the limitations by assembling a feature extraction-based generalized model that performs not only regression but also classification of RT using single-trial EEG data. The study demonstrated methods to extract temporal, spectral, and spectrotemporal features from different EEG channels [25]. The features were then fed to various regression models like Linear Regression, Ridge Regression, Support Vector Regression (SVR), Extra Tree Regression, and Random Forest Regression models for RT estimation. For classification purposes, Linear Regression Classifier, Support Vector Classifier (SVC) (with and without Stochastic Gradient Descent (SGD)), Decision Tree classifier, and Random Forest Classifier ingested the same feature set. The regression-based approach provided a root mean square error (RMSE) of 111.2 ms and a correlation coefficient (CC) of 0.74. The binary and 3-class classifier models obtained accuracies of 79% and 72%, respectively.

Though our earlier works resulted in steadfast performances, they relied on features from multiple spaces, which not only are computationally expensive but also have limited theoretical value. We felt that an approach making direct use of relevant features (as defined by previous work in neuroscience) in conjunction with state-of-the-art predictive models could achieve even better performances.

## 3. Materials and Methods

### 3.1. Experiment Details

The experiment embodied a simple reaction task requiring a response from the participants corresponding to a change of visual stimulus [26,27,28]. A computer-based interface was used to conduct the experiment, where a plus symbol (+) appeared at the center of the digital screen, and following a variable observation period, the plus symbol (+) transformed into a cross (×). The participants had to tap the space bar as soon as the symbol changed from plus (+) to cross (×). This action was repeated several times for each subject, and every single repetition represented a trial. Each participant completed a 30-minute-long experiment, with an average of 320 trials (range: 219–442) per subject.

In pursuit of generalizing our models, we tried to maximize the number of subjects while maintaining a decent balance between male and female participants. We were able to collect data from a total of 48 subjects, and our dataset is significantly larger than datasets used in comparable studies [22,23]. Among these 48 participants, 17 were female and the rest of them were male (ratio of 11:20). Also, as our aim was to find a clear relationship between EEG signals and corresponding reaction times, we targeted the younger population, who are generally healthier [29] and whose response times are less likely to be affected by various physical attributes compared to brain activities. Therefore, the ages of the participants ranged from 18 to 24 (median age 19). These participants had normal or corrected-to-normal visions, and none of them had a history of any neurological problems.

**Figure 1:**
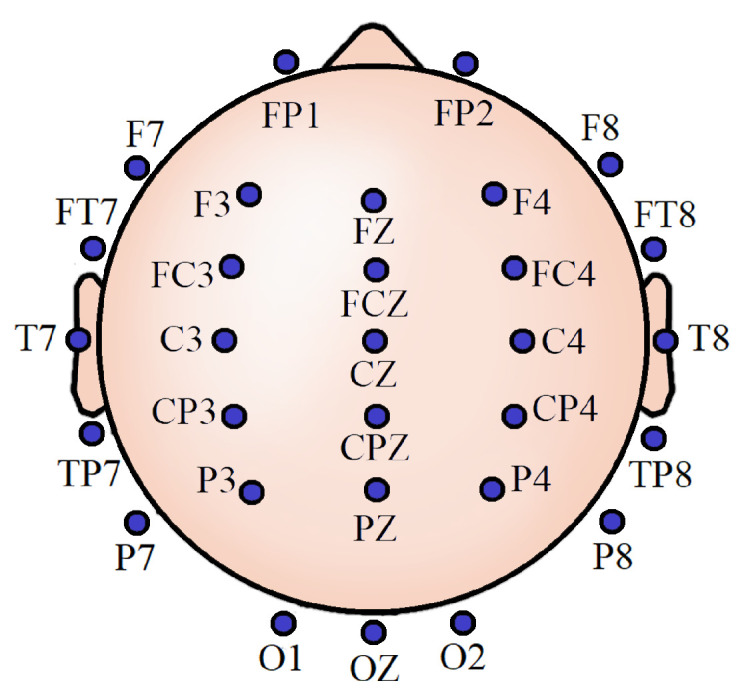
Thirty-channel electroencephalogram (EEG) scalp locations.

It has been earlier demonstrated that around 30 channels are sufficient to incorporate information from all the brain lobes adequately and to preserve analytical performance while balancing the computational requirements from both hardware and software perspectives [30]. In compliance with that, noninvasive EEG activities were recorded from 30 scalp locations for all the participants. These locations were referenced to the left mastoid, using silver/silver chloride (Ag/AgCl) electrodes attached to an elastic cap (Neuromedical Supplies Inc., Charlotte, NC，USA) and a Neuroscan SynAmps RT amplifier with Curry recording software [31]. Figure 1 shows the EEG scalp locations. All impedances were below five kΩ. We took informed consent from each of the subjects. The Institutional Review Board of Arizona State University approved all the procedures involving the experiment.

### 3.2. Data Handling and Preprocessing

EEG activities were recorded at a sampling rate of 1000 Hz. As most useful information belongs to the low-frequency region and the high-frequency region mostly contributes to noise, the recordings were filtered from DC to 400 Hz. Continuous analog-to-digital conversion of the EEG and stimulus trigger codes were performed online by the Neuroscan acquisition interface system. We performed offline data analyses using EEGLAB [32]. The continuous EEG data were downsampled to 250 Hz and filtered with a 1 to 30 Hz bandpass filter, using an infinite impulse response (IIR) Butterworth filter, and submitted to a GPU-optimized version of the infomax independent component analysis [33] procedure in EEGLAB. The ocular components in the ICA were identified using visual inspection (independently performed and then compared between two researchers), and the remaining components were back-projected to the original data, yielding unfiltered raw data that are free from ocular artifacts. The data were then re-referenced to the average of the left and right mastoid. Afterward, we normalized the data by mean and variance to remove the DC offset. In the EEG data, the starting and ending point indicators were marked for each subject. Each trial consisted of the following events that were sequentially repeated:

1. beginning of the trial (+)
2. change of symbol (×)
3. response of the subject (space bar tap)

**Figure 2:**
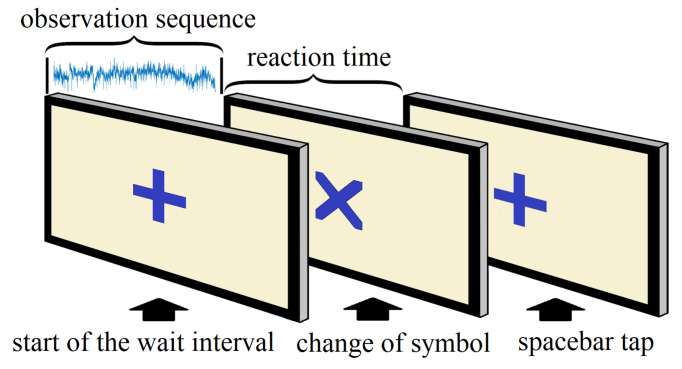
Sequential events for each trial.

The multichannel EEG signals recorded between event 1 and event 2 were the observation sequences, and the time differences between event 2 and event 3 were the corresponding RTs. Figure 2 shows the events sequentially for a single trial. We separated and saved the observation sequences and the RTs for all trials. However, RTs over 1000 ms were distinctly rare (2.6% of the total data) and we considered them exceptions. Therefore, we excluded those RTs and the corresponding observation sequences. In the end, we were left with a final dataset comprising a total of 15,324 trials from all participants. We iteratively randomized the entire dataset and split it into training and testing sets until equivalent mean, variance, and range of RTs were achieved for both sets. This also ensured proper distribution of trials from all the participants across the training and testing sets. This procedure was repeated for five different ratios of training and testing (50/50, 60/40, 70/30, 80/20, and 90/10), and finally, we settled for an 80/20 split, which provided us optimum steady performance with low variance for both training parameter estimates and testing performance statistics.

### 3.3. Periodogram

A periodogram is a straightforward tool when it comes to spectral signal analysis as it comprehensibly portrays a signal’s amplitude vs. frequency characteristics. A periodogram gives an estimation of the power spectral density of a signal. The power spectral density of a continuous function x(t) is the Fourier transform of its auto-correlation function. In other words, it is the square of the absolute valued Fourier transform of the time domain signal. However, in our case, the data is discrete and the periodogram of a discrete signal x[n] can be computed using the following equation:

(1) $$S\left(\frac{k}{NT}\right)={\left|\sum_{n=0}^{N-1}x\left[n\right]e^{\frac{-j2\pi kn}{N}}\right|}^{2}$$

where *S* is the periodogram, *k* denotes the frequency domain ordinal, and *n* represents the time-domain ordinal. *N* is the length of the sequence to be transformed, and *T* is the sampling period.

**Figure 3:**
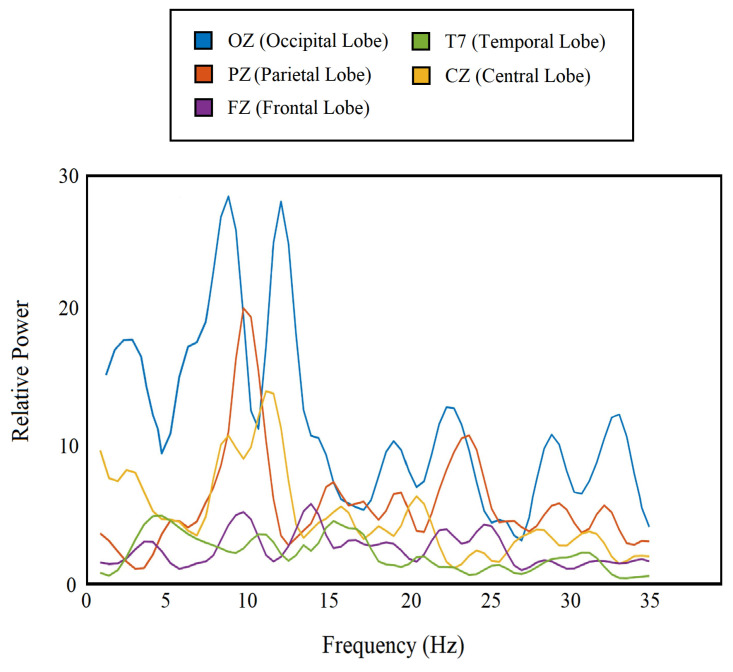
Periodogram of the single-trial EEG for 5 different channels from each lobe.

The periodogram has already aided different forms of EEG-based analyses [34]. In our study, we separated the periodogram components for frequency bands from *delta* to *beta* using a rectangular window (1–35 Hz) and used them as features. We obtained 72 feature points from each channel, which are nothing but power at 72 frequency values (1–35 Hz), and a total of 2160 (72 × 30) features from all 30 channels combined. Figure 3 shows the periodogram features for 5 different channels from 5 different lobes.

### 3.4. Deep Neural Network Models

#### 3.4.1. Fully Connected Neural Network (FCNN)

FCNN-based models are straightforward and computationally less expensive compared to most of the deep neural network architectures as they use simpler layer structures and avoid complicated mathematical operations. Hence, they are easy to implement in real-time analyses [35].

As the name implies, fully connected layers are the basic elements of our FCNN-based model. Neurons in a fully connected layer have connections to all activations in the previous layer. This layer conducts affine transformation, with matrix multiplication followed by a bias offset. The following equation shows the fundamental operation undertaken by a fully connected layer:

(2) $$Z=W^{T}.X+b$$

where **X** is the input matrix of size N × 1 and **Z** is the output matrix of size M × 1. To transform the input matrix into the output matrix, the transpose of a weight matrix **W** is multiplied by the input matrix. The size of this weight matrix is N × M. Further, a bias matrix **b** of size M × 1 is offset. All weight and bias values, starting from a random initialization, are optimized through backpropagation during training to minimize the overall loss of the model.

Rectified linear unit (ReLU) layers were also used in our model architecture. ReLU is an activation layer which introduces nonlinearities in the linear outputs of a fully connected layer. It is a simple yet critical operation that helps the model to generalize. The operation is as shown in the following equation:

(3) y=max(0,x)

where *x* is the input value and y is the output value.

Right before generating the actual class labels, a softmax layer was inserted to normalize the output of the last fully connected layer of the neural network to a probability distribution over predicted output classes. This layer performs the following operation:

(4) $$\sigma {\left(z\right)}_{i}=\frac{e^{z_{i}}}{\sum_{j=1}^{K}e^{z_{j}}}$$

where **z** is the input vector with *K* real numbers and the function normalizes it into a probability distribution consisting of K probabilities. Therefore, i=1,2,....K and $z=\left(z_{1},z_{2},....z_{k}\right)\in R^{K}$. Before applying softmax, some components of the vector might be negative or greater than one, but after implementing softmax, every single component will be in the interval (0,1) and the sum of all the components will be 1 so that they can be interpreted as probabilities.

These probabilities were then converted to class labels through one-hot encoding. In this process, the highest probability obtained among different classes for a single trial was replaced by one and the rest of the probabilities were replaced by zeros.

Cross entropy served as the loss function for our model. Cross entropy between two probability distributions *p* and *q* across an identical set of events quantifies the average number of bits required to identify an event drawn from the set if a coding procedure used for the set is optimized for an approximated probability distribution *q* instead of the true distribution *p*. Cross entropy loss function H(p,q) can be defined as the following:

(5) $$H\left(p,q\right)=-\sum_{i}p_{i}\log q_{i}$$

where the true probability $p_{i}$ is the true label and the given distribution $q_{i}$ is the predicted value of the current model.

#### 3.4.2. Convolutional Neural Network (CNN)

The CNN-based models are particularly useful where convolutional operations are needed to preserve complex inter-element information of the input matrix at the cost of higher computational requirements. They have already attained high performance in many biomedical applications and slowly started to show progress in EEG-related studies [36,37,38]. Convolutional layers perform the following operation using different filters:

(6) $$\left(h*x\right)\left[n\right]=\sum_{m=-M}^{M}h\left[n-m\right]x\left[m\right]$$

where *h* is the filter, *x* is the input, and *M* is the finite support set.

We adopted the Convolutional Neural Network architecture in our study to observe how much we can improve the performance of our model utilizing rich information extracted by the convolutional layer at the expense of the added computations. This model can be particularly useful for applications where there are computational flexibilities.

### 3.5. Binary Classification

Earlier psychological studies have shown that response times greater than 0.5 s indicate a lapse of attention in these kinds of tasks [39]. Attention lapse can affect people’s day-to-day life performances, and it becomes more crucial when it comes to critical tasks like driving, which involve a significant amount of risk. Considering the importance of it, a binary classification case was formulated by categorizing RTs into two classes [40], using RT = 500 ms as the splitting point. The class labels were as follows:

1. Fast RT (RT <= 500 ms)
2. Slow RT (RT > 500 ms)

This analysis can be valuable for qualitative comparisons, where high-accuracy categorical division is required instead of exact estimation. We designed and implemented FCNN- and CNN-based architectures to classify between fast and slow RT.

For our FCNN architecture, we installed three fully connected layers, with ReLU layers in between. The dimensions of the fully connected layers are as follows:

1. Fully Connected Layer 1:
  (a) **W1** = 2160 × 500
  (b) **b1** = 1 × 500
2. Fully Connected Layer 2:
  (a) **W2** = 500 × 100
  (b) **b2** = 1 × 100
3. Fully Connected Layer 3:
  (a) **W3** = 100 × 2
  (b) **b3** = 1 × 2

**Figure 4:**
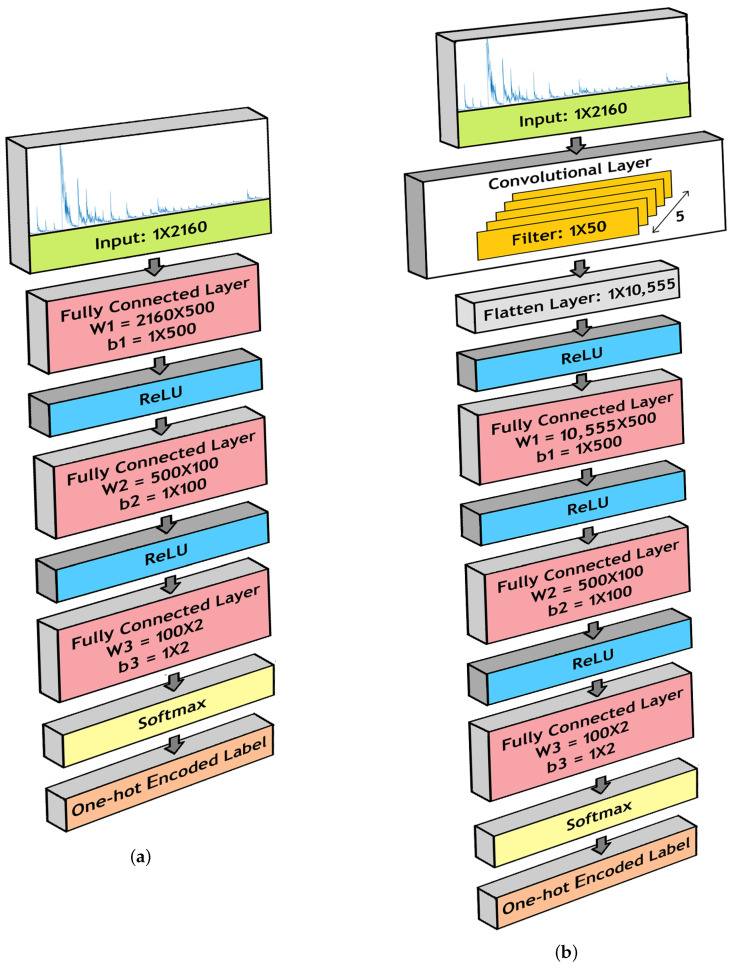
Model for binary classification using (**a**) Fully Connected Neural Network (FCNN) architecture and (**b**) a Convolutional Neural Network (CNN) architecture.

Figure 4a illustrates the model structure.

The CNN architecture for our model contained similar components as the FCNN architecture. However, before passing to the three fully connected layers, we propagated the input data through a convolutional layer. In this layer, we conducted 1-D convolution operations using 5 filters of length 50. Therefore, the dimension of the filter bank is 1 × 50 × 5. The outcomes were flattened to dimension 1 × 10,555 and passed to the fully connected layers. Figure 4b shows the CNN architecture used in our model.

### 3.6. Three-Class Classification

Studies have shown that a person’s usual response time mostly lies between 300 to 500 ms, with an average reaction time of 400 ms [41]. In our study also, the mean RT was found to be around 400 ms for all participants. We designed a three-class classification approach to analyze whether the model is able to predict more considerable variations in RT in terms of deviation from the mean. The three classes were chosen as follows:

1. Fast RT (RT <= 315 ms)
2. Medium RT (315 ms < RT <= 515 ms)
3. Slow RT (RT > 515 ms)

**Figure 5:**
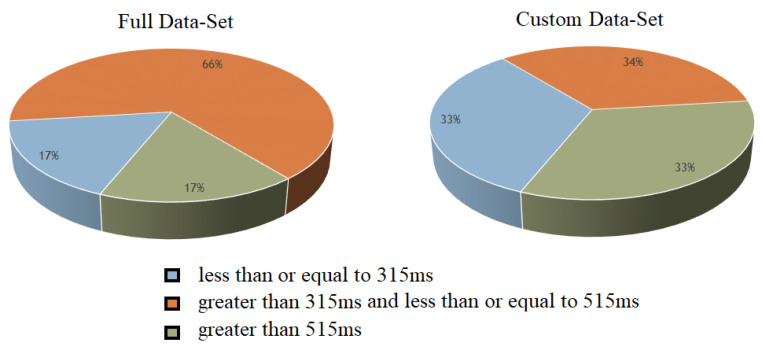
Custom dataset for 3-class classification.

The number of data points in different classes varied a lot. Notably, the medium RT class contained the most data points (66% of the entire dataset). Therefore, we properly balanced the training set beforehand, as shown in Figure 5.

**Figure 6:**
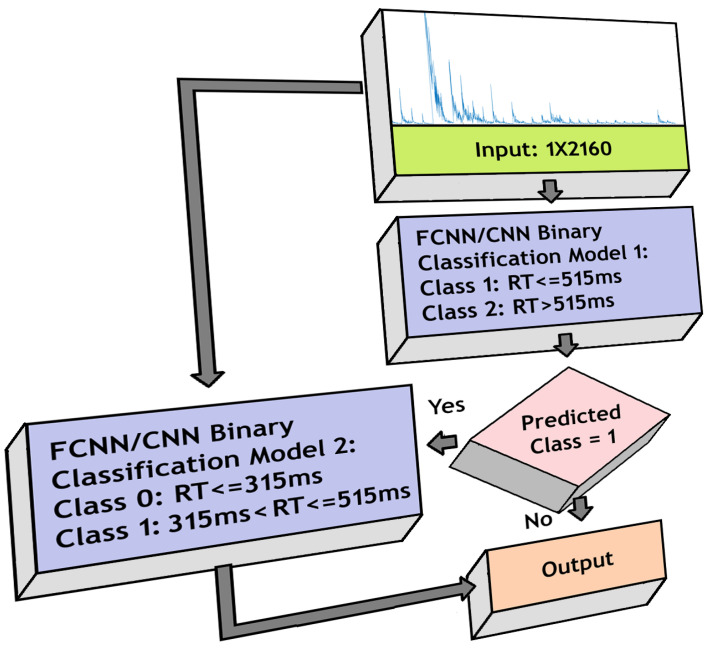
Model for 3-class classification using FCNN/CNN architecture.

We created a model combining two binary classifiers, the first classifier separated RTs using a threshold of 515 ms, and the RTs detected as higher than 515 ms were passed directly to the output layer as class 2. The ones detected as less than or equal to 515 ms were passed to the second binary classifier for further categorization into classes 0 and 1, using a threshold of 315 ms, and then were transferred to the output layer. Therefore, in the output layer, we get classes 0, 1 and 2. We again implemented FCNN- and CNN-based architectures separately and compared their performances. Figure 6 shows the model structure.

### 3.7. Regression

In our earlier approach [42], the random forest model estimated RTs more proficiently than other comparable models. Here, instead of using a single random forest model, we used two different random forest models independently trained to estimate RTs below and above 500 ms separately.

**Figure 7:**
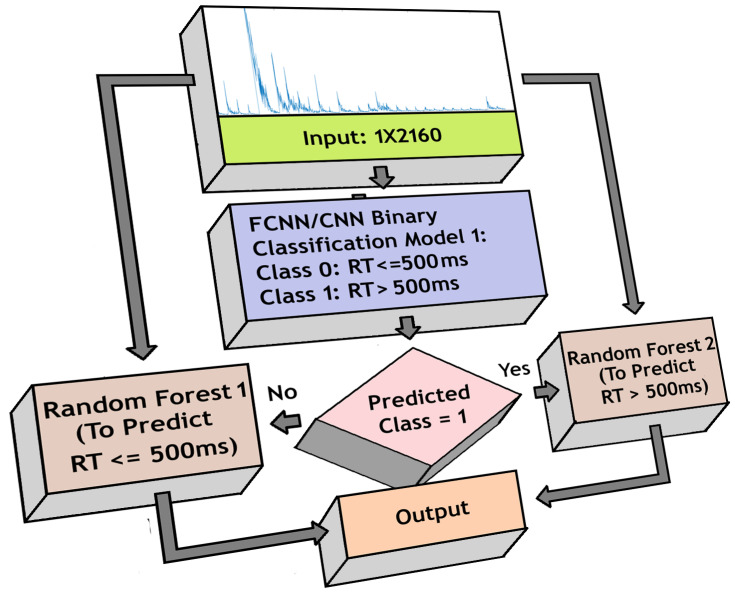
Model for regression-based reaction time (RT) estimation using FCNN/CNN architecture and Random Forest model.

We used the previously trained FCNN/CNN-based binary classifier models to initially identify if the RT is below or above 500 ms. Based on the decision, one of the random forest models was activated for each trial to generate estimation. The estimated RTs from either model was sent to the output layer. Figure 7 shows the structure used for the regression-based approach.

### 3.8. Important Channels Isolation

The classification and regression analysis helped us train and test models to predict RTs using spectral features from EEG signals. Thus, the model is able to capture both spatial and spectral features from the brain that play an important role in this sustained attention visual response task. In this part of the analysis, we use the previously trained model to isolate the significant spatial features/EEG channels. It also serves the purpose of identifying the minimum number of EEG channels required to preserve optimum model performance. The previously trained CNN-based binary classification model was used for this part.

**Figure 8:**
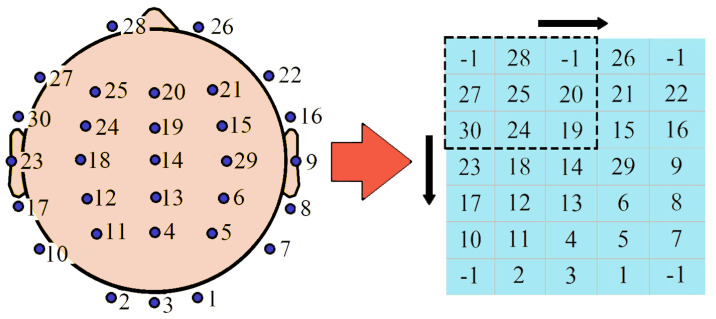
Matrix representation of the channel numbers.

To perform the task, we first created a 7 × 5 matrix that served as a 2-D topographic map, mimicking the actual positions of the EEG channels in a horizontal section of the brain. All the EEG channels were assigned a number from 1–30 based on the channel sequence we used in our data. The left part of Figure 8 shows the actual channel positions and the assigned numbers. We transferred these numbers to the 7 × 5 matrix maintaining their relevant positions, as shown in the right side of the figure. The matrix indexes without any elements were filled up with −1. After preparing the matrix, we first calculated accuracies for 30 models trained on each channel separately and identified the one most relevant channel providing maximum accuracy independently. Then, from the topographical map, we separated the 8 neighboring channels of the identified most relevant one, using a 3 × 3 matrix where the identified one was in the center. We then assessed the performance of 8 models, each trained on 2 channels (the most relevant one and one of the 8 neighbors). Based on the relative performances, we detected the second most important channel. In this manner, we kept increasing adjacent channels one at a time. We ended up with channel subsets of variable lengths and determined the highest accuracy achieved for each length. We adopted this method as our target was not to identify a random set of channels that provides maximum accuracy but to find out the most relevant region of the brain corresponding to visual stimulus-based reaction time.

### 3.9. Important Frequency Band Isolation

We used the periodogram for frequencies ranging from 1 to 35 Hz, consisting of the four major EEG frequency bands delta, theta, alpha, and beta, as shown in Table 1. In this study, we compared the contribution of these frequency bands to predict RT and to infer which frequency bands dominate in the visual response task.

Isolation of crucial frequency bands was performed using a very straightforward procedure. We trained our CNN-based binary classifier model with features from each frequency band separately and recorded the corresponding performances. Frequency bands attaining better accuracies were determined as the most important ones.

## 4. Results

### 4.1. Individual Subject Analysis

**Figure 9:**
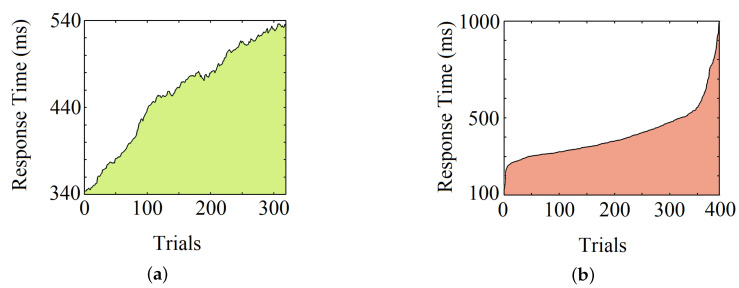
Observation of an individual RT after (**a**) the moving average operation and (**b**) the sorting operation.

Moving average operations on individual RTs show an upward trend for most participants (75%), intimating a decrease in response speed over time. Apart from that, the overall variation of RTs can be observed by sorting the RTs of a subject from low to high. The average skewness (defined by (μ−ν)/σ where μ is mean, ν is median, and σ is standard deviation) for all the subjects’ reaction time is 1.83. Figure 9a, shows the upward trend of RTs of an individual, and Figure 9b shows its overall variation.

### 4.2. Binary Classification

In this analysis, we examined the binary classification accuracy to predict RT from single-trial EEG using periodogram features. Our earlier methods used domain-specific features in conjunction with different machine learning algorithms [24]. On comparing, both FCNN and CNN-based binary classification models outperformed the previous models. While our earlier best classification accuracy was 79%, FCNN- and CNN-based models achieved accuracies of 93% and 94%, respectively. Also, both models achieved similar precisions (0.92 and 0.94, respectively) and recall (both 0.93) values.

**Figure 10:**
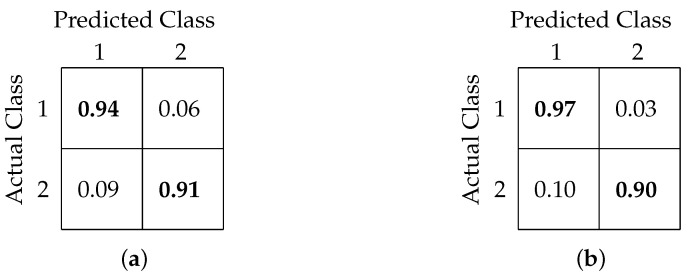
Confusion matrix for binary classification using (**a**) FCNN-based architecture and (**b**) CNN-based architecture.

Table 2 shows a comparison of classification accuracy, precision, and recall values among our former and present models. Figure 10a,b shows the confusion matrix interpretation for FCNN- and CNN-based models, respectively.

### 4.3. Three-Class Classification

Extending our analyses towards 3-class classification, we observed similar improvements in our present models. Previously, we achieved 72% of accuracy at best for 3-class classification using the Random Forest model. In our current study, we obtained an increased accuracy of 76% using the FCNN-based model, while the CNN-based model provided the best classification accuracy of 78%. We found moderately high precision values of 0.75 for both FCNN- and CNN-based models and recall values of 0.72 and 0.74, respectively.

**Figure 11:**
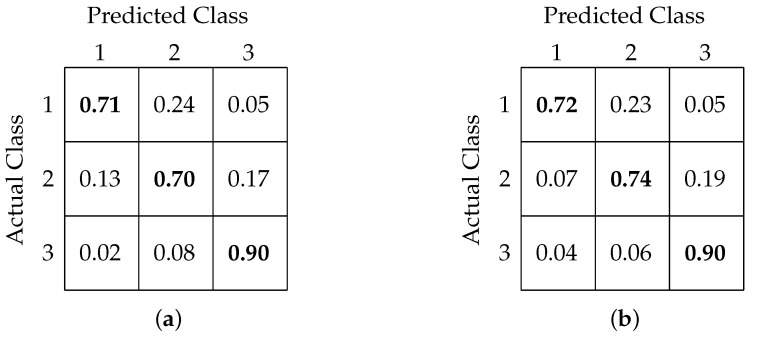
Confusion matrix for 3-class classification using (**a**) FCNN-based architecture and (**b**) CNN-based architecture.

Table 3 shows a comparison among all the models based on overall accuracy, precision, and recall. Figure 11a,b shows the confusion matrix obtained after classification using the FCNN and CNN models, respectively.

### 4.4. Regression

The primary reason to integrate the DNN-based models with the Random Forest was to utilize the previously binary classification model, which provided high classification accuracy. For both DNN-based models, predictions higher than 1000 ms were clipped to 1000 ms. The RMSE and CC obtained for the FCNN-based model were 110.4 ms and 0.78, respectively. For the CNN-based model, these values were 108.6 ms and 0.80, respectively. Clearly, the integration of DNN with our previous Random Forest model ensured better RT prediction, which earlier resulted in an RMSE of 111.2 ms and a CC of 0.74, at best.

**Figure 12:**
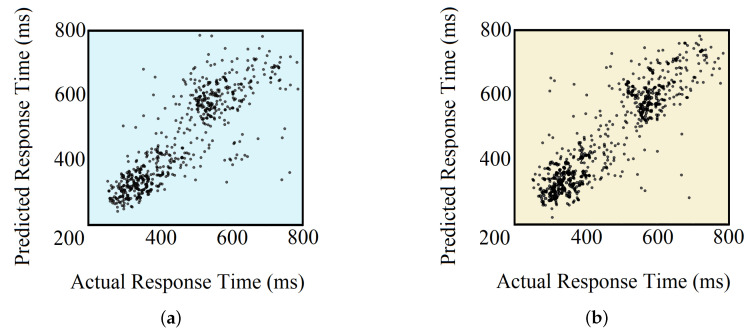
Actual vs. predicted RTs using (**a**) FCNN + Random Forest-based model and (**b**) CNN + Random Forest-based model.

Table 4 compares the performances of all the implemented methods. The actual vs. predicted RTs for the test set using both models are shown in Figure 12.

### 4.5. Important Channels Isolation

Implementing the channel isolation method, we tried to figure out the most crucial brain location relevant to visual reaction prediction and the minimum number of channels required to maintain the model accuracy. Based on our analysis, a minimum of 7 channels is needed to safeguard the model’s performance.

**Figure 13:**
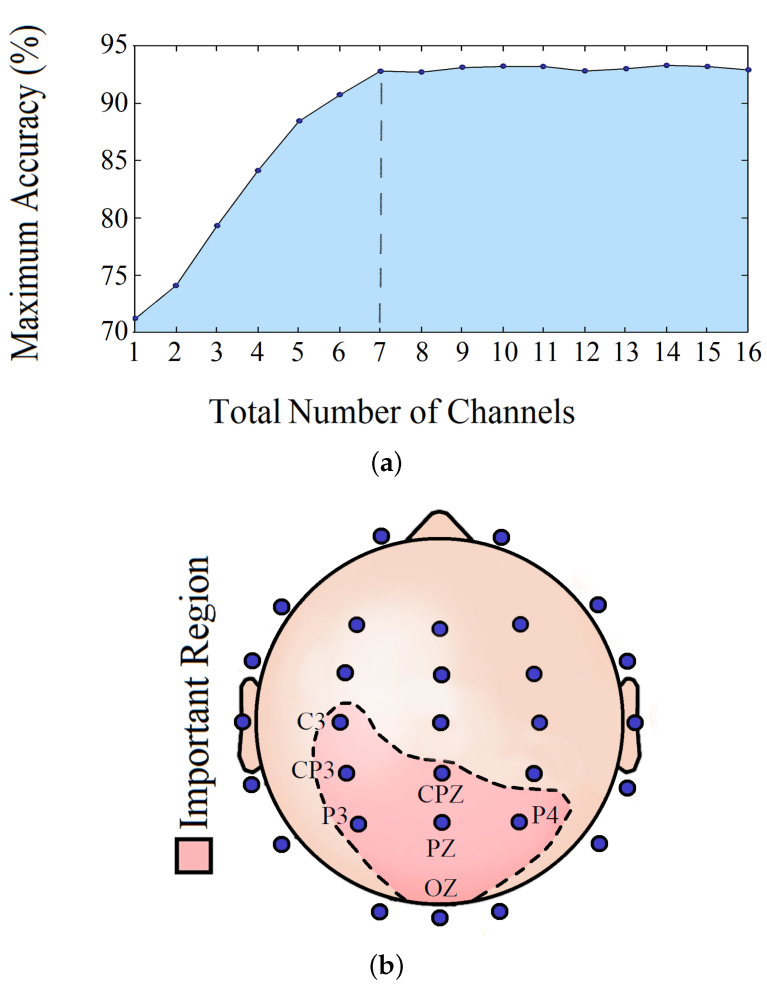
(**a**) Number of channels vs. maximum accuracy and (**b**) 7 most important EEG channels.

Table 5 shows the classification accuracy obtained every time for one or more significant channels. Figure 13a shows how the RT prediction changed with an increasing number of channels, and the steady prediction performance after the total number of significant channels reached 7. Figure 13b illustrates these relevant brain locations.

### 4.6. Important Frequency Band Isolation

**Figure 14:**
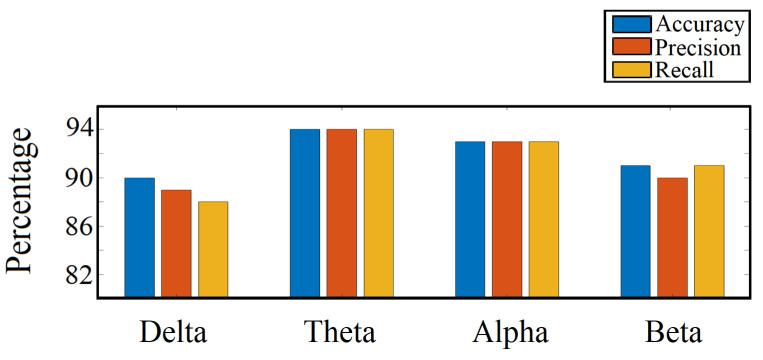
Frequency band performance comparison.

As stated in the earlier section, we trained our CNN-based binary classification model with features from different frequency bands independently and measured performance (accuracy, precision, and recall) for each. Based on this analysis, theta and alpha were the most active and dominant frequency bands in the visual response task. Figure 14 shows the relative performances we obtained.

## 5. Discussion

Analyzing the individual RTs over the time span of the experiment, we noticed that there is an overall upward trend, as shown in Figure 9a. This trend implies that a person’s average response gradually slows down, reconfirming the vigilance decrement effect generally seen in visual stimulus-based reaction experiments [43]. This effect correlates to lapses of sustained attention, which is an imperative field of interest in psychology as well as neuroscience. However, even though an average increase of RT over time prevails in most cases, it does not hold true when it comes to individual trials. RTs vary inordinately across trials, making it impossible to correlate a single RT with the time elapsed. Therefore, to analyze human reaction, it is essential to investigate the brain functionalities responsible for the action. We thus incorporated EEG signals as representatives of intricate brain information, opening doorways to analyze lapses of attention and to predict RTs.

The DNN models introduced in our study utilized spatial features in the form of EEG channels along with spectral features in the form of a periodogram of the EEG signals. The integration of both categories ensured maximum utilization of available information and thus resulted in stellar performances. Nevertheless, the major limitation of the existing EEG data acquisition process is the presence of human- and machine-related artifacts. Although EEG is considered the gold standard for BCI, it does not capture intricate and minute details relevant to various mental states. Human-related errors or noises are the primary reasons most psychology studies are skewed. More subject-specific investigations can help to assess these errors and to determine ways to improve data-capturing and -filtering methods.

In our binary classification approach, the primary target was to identify the lapses of attention, which are defined as RTs greater than 500 ms from a psychology point of view. Our proposed CNN-based model could predict these lapses with a considerable high accuracy of 94%, based on prior EEG data from a single trial. This reconfirms the fact that EEG signals contain sufficient information to predict response time, and this information can be used to alert the person about forthcoming attention lapses in real-time, once implemented in wearable hardware.

Our three-class classification analysis aimed to test whether the classification models are able to predict the critical variations in RTs. The two extreme classes, RT less than 315 ms and greater than 515 ms, were classified with accuracies of 72% and 90%, respectively, while the in-between class was classified with 74% accuracy. From these results, it is clear that the classification models were able to identify slow RTs with the highest accuracy. The confusion matrices in Figure 11 show that there was a maximum confusion between the fast and medium RT classes and that around 23% of the fast RTs were predicted as medium RTs.

Finally, we performed regression-based analyses for the quantitative prediction of RTs. In order to do that, we came up with a novel approach by fusing both DNN-based binary classification models with random forest regression models. These DNN-based models exceeded our earlier models by a fair margin in terms of performance. Our CNN-based model was able to achieve a correlation coefficient of 0.8 and RMSE of 108.6 ms, while other contemporary studies resulted in a maximum CC of 0.61 [22] and a minimum RMSE of 119.5 ms [23] for subject-specific analysis. This indicates the distinction of our model, and the narrow confidence line manifested in the actual versus predicted RT plot (Figure 12) supports the argument. However, from the aforementioned figure, two clusters of RTs can be seen, which are expected as the result of using binary classifiers on the top of regression models. This can be further improved by designing a DNN-based direct regression model that can achieve comparative performance. On another note, as regression is comparable to classification, where the number of classes is equal to the number of all possible outcomes, our regression-based analysis ensures the feasibility of *N*-class classification (where *N* > 3) with acceptable performance, using our architectures.

We tried to answer some of the pertinent questions by analyzing which EEG channels and frequency bands can play an imperative role in sustained attention tasks. From the channel isolation results, we can see that the left central (C3, CP3, and CPZ) along with parietal (P3, PZ, and P4) and occipital (OZ) lobes were the most dominant locations for RT estimation in the visual response task. The significance of the left central lobe found in our study agrees with our earlier findings and the fact that most of the subjects were right-handed, indicating more response from the left part of the brain. Furthermore, the parietal lobe plays a significant role in the visual attention tasks, evident by earlier studies [44]. The inclusion of the occipital lobe is justifiable, considering that it is the visual processing center of the mammalian brain [45]. In Table 5, it can be seen that, by analyzing channel CP3 only, we can make a meaningful prediction about RT. The prediction accuracy got better as other significant channels were added to the analysis. However, in Figure 13a, it is shown that the prediction accuracy remained almost the same as more channels were added after the 7th significant channel CPZ. On analyzing the dominant frequency bands, we found that theta and alpha bands were the most active ones. Our frequency band analysis complies with other experimental studies, where it has already been shown that alpha and theta oscillations play a significant role in sensory tasks related to sustained attention and mental fatigue [46,47]. Therefore, holistically, it can be inferred from our study that the alpha-theta oscillations in the left central, parietal, and occipital lobes are crucial in performing visual reaction tasks.

## 6. Conclusions

This paper presented methods to predict human RT in a visual response task from information embedded in their EEG signal. Generalized DNN-based models were trained with periodogram features from EEG to predict RT of a single trial. The sizable dataset containing more than 15,000 trials from 48 subjects combined with state-of-the-art estimation models corroborated our work’s reliability and viability. Furthermore, simplified and straightforward DNN architectures ensured fast and efficient computations. Significant improvements in the classification of slow and fast RTs were noticed, achieving an accuracy of 94%. The accuracy of the 3-class classification also increased from 72% to 78%. Furthermore, the regression-based approach provided better RT prediction, attaining a CC of 0.8 and RMSE of 108.6 ms. Our channel selection method showed that the performance could be preserved with just 7 EEG channels, which further reduces the cumulative computational requirements. This realization provides future possibilities of implementing the entire system in hardware and predictomg RT real-time.

This is one of the few studies where human RT is predicted using features from EEG in sustained attention-based visual response task. Accurate prediction of RT helps precise assessment of the mental state, which benefits not only researchers in the field of psychology and neuroscience but also CI applications. The generalized model can help to monitor people with all kinds of neuromuscular disorders and can help them in rehabilitation. In that case, more data collection is required to test the model in patient-related studies and to have a better understanding of the spatial and spectral characteristics that regulate RT.

As reaction time is a combination of perception, processing, and response, estimation of reaction time can be improved if we take into account other factors like eye movement, muscle memory, or emotional parameters [48]. Furthermore, we can extend this study to a more subject-specific level to answer more psychology-related questions [49,50,51]. Similar experiments involving different visual tasks, levels of complexity, and response type may reveal new information about the relationship between EEG signal and reaction time. Apart from that, reaction times in practical situations can result from multiple concurrent stimuli (like a combination of auditory and visual stimuli). Therefore, it is worth analyzing the impact of multiple stimuli on EEG signals and reaction times. Simultaneously, exploring other relevant methodologies, algorithms, and models may further improve the overall performance and may reduce computational loads. We are looking forward to continuing our research and to untangling the secrets revolving around the human brain little by little with our upcoming works. 

## Figures and Tables

**Table 1:** Frequency bands.

Frequency Band	Frequency Range (Hz)
delta (δ)	1–4
theta (θ)	4–8
alpha (α)	8–12
beta (β)	12–35

**Table 2:** Binary classification results.

Algorithm	Accuracy (%)	Precision	Recall
Linear Regression	67	0.67	0.67
Decision Tree	63	0.63	0.63
Support Vector Classifier (SVC)	73	0.72	0.73
Stochastic Gradient Descent (SGD) + SVC	63	0.63	0.63
Random Forest	79	0.79	0.78
FCNN	93	0.92	0.93
CNN	94	0.94	0.93

**Table 3:** Three-class classification results.

Algorithm	Accuracy (%)	Precision	Recall
Linear Regression	52	0.54	0.53
Decision Tree	56	0.55	0.56
Support Vector Classifier (SVC)	70	0.54	0.59
Stochastic Gradient Descent (SGD) + SVC	59	0.56	0.56
Random Forest	72	0.71	0.70
FCNN	76	0.75	0.72
CNN	78	0.75	0.74

**Table 4:** Regression-based estimation results.

Algorithm	CC	RMSE (ms)
Linear Regression	0.56	158.7
Ridge Regression	0.56	157.6
Support Vector Regression (SVR)	0.60	136.7
Extra Tree Regression	0.73	114.4
Random Forest Regression	0.74	111.2
FCNN + Random Forest	0.78	110.4
CNN+ Random Forest	0.80	108.6

**Table 5:** Isolated channels.

Channel/Channels	Accuracy (%)
CP3	72.2
CP3, C3	73.8
CP3, C3, P3	79.1
CP3, C3, P3, OZ	84.1
CP3, C3, P3, OZ, P4	88.3
CP3, C3, P3, OZ, P4, PZ	90.8
CP3, C3, P3, OZ, P4, PZ, CPZ	92.7
